# Impact of projected climate and socioeconomic scenarios on state-wise annual dengue incidence in India using ensemble models

**DOI:** 10.1371/journal.pntd.0014159

**Published:** 2026-03-31

**Authors:** Avik Kumar Sam, Ipsita Pal Bhowmick, Harish C. Phuleria

**Affiliations:** 1 Environmental Science and Engineering Department, Indian Institute of Technology Bombay, Mumbai, India; 2 Indian Council of Medical Research – Regional Medical Research Centre, Dibrugarh, India; 3 Centre for Climate Studies, Indian Institute of Technology Bombay, Mumbai, India; Basque Center for Applied Mathematics: Centro Vasco de Matematicas Aplicadas, SPAIN

## Abstract

India, the world’s most populous country, has reported over 1 million dengue cases and ~3,000 deaths between 2007 and 2022. With the annual state-wise data, we examined the spatiotemporal distribution of dengue in 28 states and eight union territories for 16 years across India. Using state-wise data on climatic variables, socio-economic inequities and land-use land-cover changes, potential determinants for the state-wise transmission were identified through a supervised regression model. The identified determinants were then mapped to various novel developmental scenarios, which were designed based on the existing shared socio-economic pathways. To estimate the dengue burden for each scenario, ensemble models of XGBoost and Gradient Boosting regression algorithms were developed. We note that 73% of the cases occurred between 2016 and 2022, highlighting a significant increase in dengue outbreaks across the country. All Himalayan states, which witness colder temperatures, have witnessed a growth in cases: Himachal Pradesh reported 168 times more cases between 2016 and 2022 than those observed between 2007 and 2015. The models suggest that dengue incidences may potentially change under future socioeconomic burden, although projections are associated with substantial uncertainty and should be interpreted as potential trajectories rather than definitive forecasts. We estimate that development focused on sustainability (874.2 per 10 million; 95% CI: 535.4, 1212.9) and fossil fuels (888.02 per 10 million; 95% CI: 521.2, 1254.9) will relatively cause a lesser burden across the country by the 2030s. Southern states are projected to have higher dengue outbreaks, while Jharkhand, a historically malaria-endemic state, is estimated to report twice as many cases in 2050 as what was reported in 2022. Given the uncertainty associated with long-term projections, public health strategies may benefit from adaptive approaches which are backed by climate- and socioeconomic-data integrated early warning systems that can respond to evolving climatic and socioeconomic conditions influencing dengue transmission. Our study provides insights into how the spread of dengue will change with varying models of socio-economic development, which highlights the spatial heterogeneity in potential future dengue risk, suggesting that resource allocation and surveillance efforts may benefit from region-specific prioritisation instead of a uniform policy.

## Author summary

We study the impact of climate change on dengue cases in Indian states using machine learning models. With modified socio-economic development scenarios, we projected the increase in dengue cases for each state in a near (2030) and mid-scenario future (2050). Historically, dengue cases reported during 2016–2022 were three times higher than those reported between 2007–2015. Our modelling results suggest that dengue incidence could change under different future socioeconomic pathways. However, the magnitude and direction of these changes vary across scenarios and are associated with considerable uncertainty, highlighting the need for cautious interpretation of long-term projections. In the immediate future, development focused on sustainability will likely have a relatively lesser increase, while a regional rivalry scenario may witness the highest increase. In the southern India, increased outbreaks are estimated in the future. In contrast, dengue is likely to decrease significantly in the densely populated states of the Gangetic Plains. The projected spatial heterogeneity in dengue incidence suggests that targeted surveillance and early detection systems may be particularly important in states where future risk is projected to change. Further, integrating climate and socioeconomic information into routine monitoring could improve preparedness for potential changes in transmission patterns.

## 1. Introduction

Dengue, an arthropod-borne viral infectious disease, is prevalent and endemic to more than 100 countries across the world [1,2,3]. Dengue incidences have drastically multiplied, with 5.2 million cases reported in 2019, which is ten times more than that reported in 2000 [4]. Previously, global models have estimated an annual 390 million dengue infections with 24% clinical manifestations [5] and 3.8 billion people inhabiting the regions suitable for dengue transmission [6]. Around 70% of the global burden is contributed by the Asian countries, while South American and Western Pacific regions are also severely affected [4].

India, located in South Asia, is the world’s most populous country with a very high population density [UN, 2024]. The large geographical spread situated between 68°7′ E to 97°25′ E and 8°4′ N to 37°6′ N divides the country into six major zones, varying from alpine in the north to arid in the west and tropical humid regions in the southwest and island regions [7,8]. Since the onset of the 21^st^ century, dengue has emerged as the major vector-borne disease, as epidemics are frequently reported across the country [9,10]. Previously, dengue was primarily restricted to urban areas, but the geographical expansion of dengue epidemics has been frequently reported in semi-urban and rural India [11,12]. Changing lifestyles aided by rapid urbanisation, climatic change, population growth, migration, and climate change were attributed as strong drivers of the frequent dengue outbreaks [13,12].

Further, dengue transmission is highly sensitive to climatic conditions, particularly temperature, rainfall and relative humidity. Studies have revealed a link between increased climatic suitability for transmission and expansion of regions [14,15]. The changing climate is expected to have a catastrophic impact on the socio-economic hierarchy found in these climate zones [16,8]. The true impact of a conjunction between climate change and socioenvironmental factors on dengue transmission and the associated risk in India is unknown [17]. The epidemiological triangle of dengue includes interactions between pathogen, hosts, and mosquito vectors, i.e., *Ae. aegypti* and *Ae. albopictus*, along with their respective responses to the environment. As the virus undergoes maturation and development in the mosquito vectors transmitting the disease, dengue becomes sensitive to climate change [1,2,3]. Moreover, the life cycle of the major carrier of the dengue virus, i.e., *Ae. aegypti* is directly influenced by ambient temperature and rainfall [17,14].

There is growing evidence pointing out the association of dengue epidemics with temperature, rainfall, and relative humidity. Various modelling approaches were used for studying the impact of climate change on dengue transmission and risk [18,19,20]. More recently, machine learning models have been developed to forecast dengue in Colombia [21], Brazil [22], Malaysia [23], China [24], Philippines [25] and in Pune, India [26]. Dengue predictive models based on machine learning have been extensively used in Latin America for informing public policy [27]. Additionally, data on the prevalence of comorbidities and indicators of socio-economic development remain insufficiently explored in India despite their relevance. However, the limited availability of detailed data on dengue further complicates efforts to analyse its patterns and identify the covariates responsible for increased vulnerability. As dengue cases continue to rise rapidly and the country witnesses a shift from malaria to dengue [28], conducting a national-level study, though ecological in nature, becomes crucial to better understand the extent of dengue transmission and identify potential determinants across the country. In this paper, we analyse the spatiotemporal distribution of dengue incidences and deaths across India in the last 16 years. We model the potential link between the dengue outcomes and weather and socio-economic variables and estimate their short- and long-term distribution in 2030 and 2050. We suggest potential policy mandates for the Indian government to mitigate the risk associated with dengue.

## 2. Methodology

**Ethics statement**: Not required as the study is based on state-wise aggregated data, not individuals and the aggregated disease data is obtained from NCVBDC, a publicly accessible database.

### 2.1. Study design

India is divided into 28 states and 8 union territories, and each region has a comprehensive public health system with well-equipped health centres. These centres are connected under the Integrated Disease Surveillance Programme (IDSP) and the National Vector Borne Disease Control Programme (NCVBDC). Further, the Department of Health Research and the Indian Council of Medical Research (ICMR) have a well-established network of 134 virus research and diagnostic laboratories spread across the country that have conducted more than a million diagnostic tests [29,30].

With a population of 1.2 billion spread across 3.28 million km^2^, as per the last official census conducted by the Central Ministry of Statistics and Programme Implementation in 2011 [31], it is difficult to conduct a longitudinal or cross-sectional study focused on the entire country, which is both resource-intensive and cost-exhaustive. Further, India has a diverse socio-economic hierarchical structure and highly varied climatic zones, which makes it further difficult to separately assess the underlying determinants responsible for dengue transmission across the country. In contrast, an ecological study design offers an efficient approach to identifying population-based patterns, providing a valuable benchmark for future cross-sectional or longitudinal studies at smaller spatial scales [32,33].

For the present study, annual data on clinically reported dengue cases and deaths for each state were obtained from the NCVBDC for 2007–2022. We included all states but removed the years 2007–2010 for modelling, as several states reported zero cases in the same period due to an underdeveloped diagnostic network. The Indian Council of Medical Research and the Department of Health Research, Government of India, strategised the establishment of a network of virology diagnostic laboratories in 2009–2010 [30]; thus, testing for dengue detection was also limited. Moreover, the excessive zeroes present in the datasets often lead to biased splits, which reduce the overall performance of the model by hindering adequate fitting, causing under- or overestimation, and consequently reducing the accuracy of the predictions [34,35].

### 2.2. Covariates

#### 2.2.1. Meteorological data.

The humidity, precipitation, moisture, and temperature data were obtained using the Modern-Era Retrospective Analysis for Research and Applications version 2, or MERRA-2. The MERRA-2 provides the monthly advanced atmospheric reanalysis data from the National Aeronautics and Space Administration’s Global Modelling and Assimilation Office (GEOS) at an approximate resolution of 0.5° × 0.625° [36]. The MERRA-2 data is sourced from the GEOS atmospheric data assimilation system and the GSI analysis scheme. The finite-volume dynamical core that uses a cubed-sphere horizontal discretisation at 72 hybrid-eta levels from the surface of 0.01 hpa is applied for further computation on a longitude-latitude grid at the same spatial resolution using a 3DVAR algorithm. This algorithm is also based on the GSI scheme with an update frequency of 6-h and incorporates the FGAT technique for temporally accurate calculations of observation-minus-background departures. More information about the MERRA-2 reanalysis data can be found elsewhere [36].

We considered surface temperature, soil moisture, precipitation, and specific humidity for the present study. There is growing evidence pointing out the association of dengue epidemics with temperature, rainfall, and relative humidity [18,19]. Hence, the monthly level data was aggregated temporally to the annual observations, while it was spatially restructured into states and districts using pre-defined boundaries of geographical coordinates. From the gridded data, annual maxima and minima were calculated for each state, while the total precipitation was estimated separately.

#### 2.2.2. Socio-economic data.

For the present study, we considered the National Family Health Survey (NFHS), a comprehensive survey conducted by the government of India that had multiple rounds of surveys in representative household samples. The NFHS is conducted by the International Institute for Population Sciences for the Ministry of Health and Family Welfare, with a focus on providing national-level information on the quality of life, health and planning services, family planning, child and maternal health, nutrition, reproductive health, and disease-specific information such as anaemia. The NFHS seeks to form a background for policy legislation across India while highlighting critical insights into health and socio-economic indicators. The data collected as part of the NFHS is primarily through interviews and physical measurements, which makes it a reliable and robust source of information. The NFHS 4 was conducted between 2015 and 2016, while the NFHS 5 was organised between 2019 and 2020 (MoFHW, 2023).

For the present study, we considered the years 2010 – 2016 as representative of NFHS 4 and the period 2017 – 2022 as representative of NFHS 5, to deal with the gaps created by the lack of surveys during the study period. From the NFHS, variables related but not specific to nutrition, health and household were considered. The household recode file, which consists of data for each household, and the members recode file, which has data for each household member, were used. The indicators were extracted from the raw data files, and preprocessing was performed for quality checks and analysis. The individual data was aggregated into states, and the final database was prepared for application in developing mathematical models. The broad categories of the parameters sourced from NFHS is provided in Table A in S1 Appendix.

#### 2.2.3. Land-use land-cover data.

We used the decadal land use and land cover (LU/LC) changes at 100m resolution derived from Landsat 4 and 5 Thematic Mapper for the present study. More information can be found elsewhere (ROY, 2016). From 2016 onwards, we used the satellite data from the ESA Sentinel-2 imagery that provided the data at a 10m resolution [37]. We combined both datasets using standard definitions and extracted the individual areas for water bodies, flooded vegetation, bare and built-up areas, snow cover, forests, and croplands. For the present study, the base maps used in the spatial analysis were obtained from the official Online Maps Portal, maintained by the Ministry of Space and Technology, Government of India [38].

### 2.3. Modelling

#### 2.3.1. Data pre-processing.

For the present study, we combined the state-aggregated socio-economic, land-use land-cover, meteorological and dengue incidence data after ensuring that all the state names are consistently mapped across both spatial and temporal resolution. Due to a skewed distribution present in the reported cases, we applied logarithmic transformations to the reported cases to stabilise variance and reduce skewness. Post-data cleaning, we transformed the data using the Quantile Transformer, which is highly robust to the influence of outliers [Amorim et al., 2023].

The source of the dengue and the covariate data is provided in Table 1.

**Table 1:** Description of the data used in the present study.

Variables	Source	Spatial	Temporal Level	Link	Reference
Dengue	NCVBDC	States	Annual	https://ncvbdc.mohfw.gov.in/	
Socio-economic	NFHS 4	Individual (aggregated into States)	2015-16	https://dhsprogram.com/methodology/survey/survey-display-355.cfm	
	NFHS 5	Individuals (aggregated into States)	2019-21	https://dhsprogram.com/methodology/survey/survey-display-541.cfm	
Land-use land-cover	Landsat 4 and 5 Thematic Mapper	100 m		https://www.earthdata.nasa.gov/data/catalog/ornl-cloud-decadal-lulc-india-1336-1	Roy, 2016
	ESA Sentinel-2 imagery	10 m		https://livingatlas.arcgis.com/landcover/	[37]
Meteorological	MERRA-2	States	Monthly (Annual after aggregation)	https://giovanni.gsfc.nasa.gov/giovanni/	[36]

#### 2.3.2. Identification of potential determinants.

We first developed a supervised stepwise regression model, where variables were included if their p-value was significant at the 95% confidence level and the adjusted R-squared improved by at least 1%. At the same time, variables were excluded if their p-value exceeded 0.05. This process was repeated until all remaining variables were statistically significant. The generalised regression model can be written as,


$$\text{log}Cases_{normalised}=\alpha +\beta_{i}.{\left\{Meteorology\right\}}_{i}+\gamma_{j}.\{{LULC\}}_{j}+\delta_{k}.{\left\{Socioeconomic\right\}}_{k}+year+\varepsilon$$

where the log $Cases_{normalised}$ denote the natural logarithm of the population-normalised cases, and i,j,k represent the different meteorological, land-use land-cover and socio-economic covariates, respectively. The dengue incidence values were log-transformed to stabilize variance. As some state-level observations included zero values, a *log (x + 10)* transformation was applied to avoid undefined values associated with *log (0)* while retaining all observations in the dataset. A Box–Cox transformation analysis was conducted to assess the appropriate transformation of the incidence data. The estimated λ value (≈0.11) indicated that a logarithmic transformation was appropriate; therefore, a *log (x + 10)* transformation was used [39]. The year variable reduces bias from temporal autocorrelation and ensures that the time-dependent trends are explicitly modelled.

For our final model, we used data from 2010 onwards and merged the five northeastern states of Arunachal Pradesh, Manipur, Mizoram, Meghalaya, Tripura and Sikkim, as our model was sensitive to these states. Further, these states have small land areas and have been historically classified as malaria hotspots where the health infrastructure was primarily focused on malaria control [40]. However, frequent dengue outbreaks have been reported from these regions from 2010 onwards as improved dengue testing also occurred simultaneously [41,42, 43,44].

We used the Variable Inflation Factor to check for multicollinearity among the selected predictor variables and then also performed residual diagnostics tests, such as Kolmogorov-Smirnov and Jarque-Bera Tests. Visual representation of the spread of residuals was performed to confirm the randomness and normality of the residuals. The regression model was also further validated by a five-fold cross-validation approach, which explained the generalizability of the model.

#### 2.3.3. Defining socio-economic scenarios.

**Fig 1:**
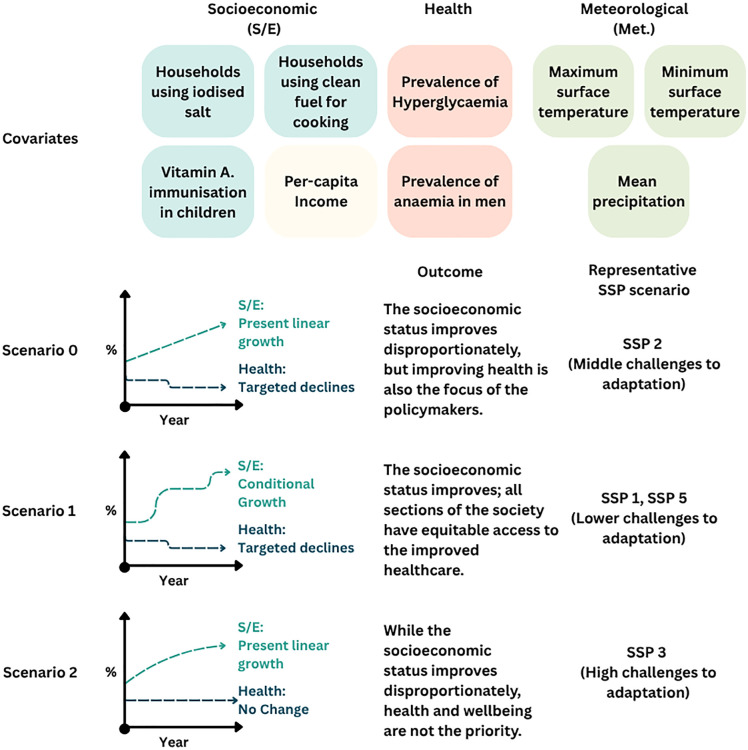
Scenarios defined for the health and socio-economic covariates and their mapping with the SSPs.

For the prediction modelling, we defined three hypothetical scenarios: Scenario 0 or S0, Scenario 1 or S1, and Scenario 2 or S2. We identified two types of covariates from the association model: Socio-economic (SE) covariates and Health covariates. For each type, we projected the differential growths of the covariates. As there was a proportional increase in the SE covariates, as we observed from the NFHS data, we considered a linear growth for these covariates for both S0 and S2. For Scenario 1, we used conditional growth for the states based on the assumption that the government will focus exclusively on the states having lesser prevalence for each covariate with targeted schemes, while the remaining states having higher prevalence, will continue to witness linear growths till the time they achieve 99.9% prevalence. For the health covariates, we observed that the prevalence has decreased; hence, targeted declines were assumed for both S0 and S1, while the present values were taken for S2. The high levels of socio-economic development in the S0 and S1 will lead to an increased decline in the prevalence of these covariates; while the SE status improves in the S2, health and general well-being are still not the priority. Overall, S1 represents the best scenario for holistic SE development and health improvement, followed by S0 and then S2. We then mapped each scenario with the defined Socio-economic Shared Pathways or SSPs [45,46,47,48] based on the national-level One Health scenarios of the Netherlands [49]. The SSPs have been briefly summarised in Table B in S1 Appendix, while the mapping is provided in Fig 1.

Additionally, we projected the data for meteorological variables such as maximum and minimum surface temperature, mean precipitation, and per-capita income corresponding to each SSPs. We used the estimated changes for the meteorological variables for the river basins spanning across the country [50]. Mishra et al. [50] have developed a bias-corrected dataset for four SSP scenarios, namely SSP126, SSP245, SSP370 and SSP585, using 13 General Circulation Models from Coupled Model Intercomparison Project-6. We projected these predictions on the administrative boundaries of the states and union territories of India. Similarly, the projections for the annual change in GDP and population were used for each of the SSP scenarios [51].

#### 2.3.4. Developing an ensemble model for predictions.

Similar to the association model discussed in Section 2.3.1, the natural logarithm of population normalised cases was taken as the dependent variable, while the covariates discussed in Section 2.3.2 constituted the independent or the predictor variables. We then employed two sophisticated machine learning models – XGBoost and Gradient Boosting (GB) for capturing the complex relationships between the covariates and the dengue cases. These models together can capture the non-linear patterns in the data [52]. To address the potential risk of overfitting given the modest sample size, we implemented several safeguards. Both Gradient Boosting (GB) and Extreme Gradient Boosting (XGBoost) inherently incorporate regularization mechanisms that constrain model complexity. For GB, hyperparameters such as ‘max_depth,’ ‘min_samples_split,’ ‘min_samples_leaf,’ and subsample act as implicit regularizers. For XGBoost, additional explicit regularization terms (λ: L2, α: L1) were included in the hyperparameter search space.

Instead of manually tuning the hyperparameters, we automated the search for optimal model configurations by performing Bayesian Optimization with 5-fold cross validation [53,54] This allowed us to balance model flexibility with generalization. The inclusion of parameters such as learning rate, maximum depth, subsample ratios, and column sampling ensured that models were not only tuned for predictive accuracy but also constrained to avoid excessive complexity.

We integrated the best models after Bayesian Optimization into an ensemble model using a Voting Regressor, which combines their predictions to enhance accuracy. This approach again helps to minimise overfitting by leveraging the unique strengths of each model, resulting in a more robust and generalisable model across different datasets. We assessed the model’s performance using metrics such as the R² score, and mean absolute error (MAE). We obtained an R^2^ score of 91.7% and MAE of 0.5, while on transforming the cases back into their original form, an R^2^ score of 76.8% and MAE of 624.8 was achieved. We also applied an 8-fold cross-validation approach for validating the robustness of the model, for which the data was divided into multiple subsets and the model was trained and tested across different subsets. We obtain an average R^2^ score of 0.66, which indicates that the model is generalisable and performs consistently well, minimising the risk of overfitting. This ensures that the model is accurate in historical cases and well-suited for making predictions in future scenarios.

**Fig 2:**
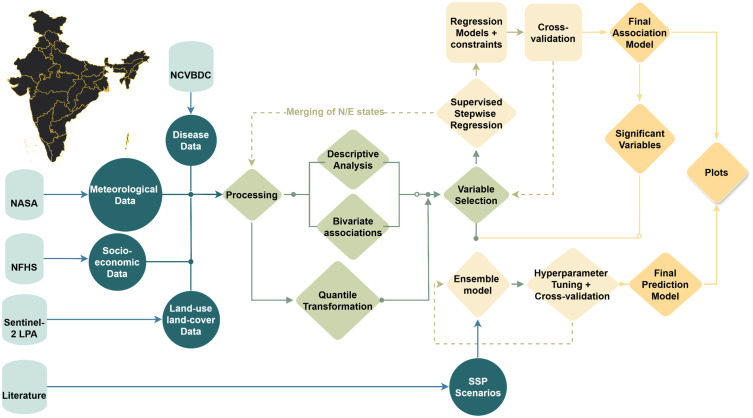
Methodology framework illustrating various inputs, outputs and data processing. Source of base map: Official Online Maps Portal, maintained by the Ministry of Space and Technology, Government of India. (https://onlinemaps.surveyofindia.gov.in/Digital_Product_Show.aspx). The terms for usage can be viewed here: https://www.surveyofindia.gov.in/pages/copyright-policy.

We then used the ensemble model to generate predictions for the different scenarios discussed above in Section 2.3.2. The model uncertainty was quantified using the Mean Absolute Percentage Error, which measures the overall deviations of predicted values from the actual cases without any logarithmic transformations. Additionally, the uncertainties in the input meteorological covariates were incorporated into the overall uncertainty, resulting in a final estimated uncertainty of 19.5%. Finally, we employed a leave-one-out cross-validation approach, where each state and year was removed individually to identify the most influential states and the specific years to which the model is most sensitive. From the final ensemble model, the feature importance was also estimated for each determinant to assess its importance in the predictions. The XGBoost model assigns the feature importance based on split gains, whereas, the GB model assigns the importance based on reduction in variance. The modelling was done on Python (v. 3.10.6) using libraries including Statsmodels (v· 0·13·2), Scikit-Learn (v·1·1·2), and Scipy (v. 1·13·0). The overall methodological framework is provided in Fig 2.

## 3. Results and discussion

### 3.1. Spatial distribution of dengue in India

**Fig 3:**
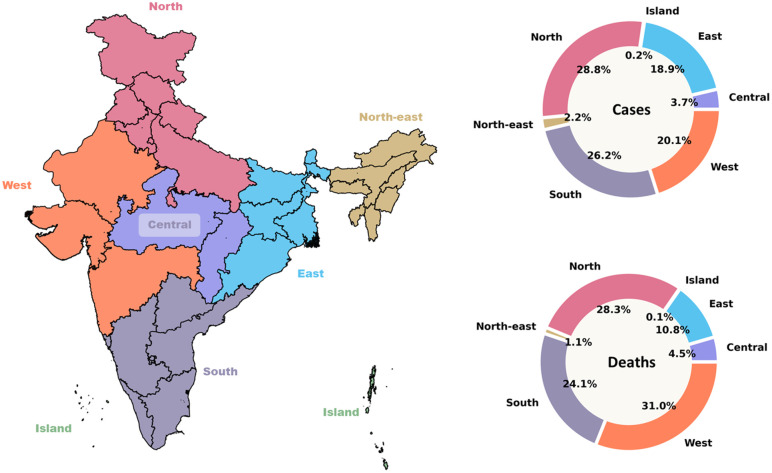
Proportion of dengue cases and deaths across the seven zones between 2007-2022. The map on the left show the states and union territories, which are colored by the zones they represent. Source of base map: Official Online Maps Portal, maintained by the Ministry of Space and Technology, Government of India (https://onlinemaps.surveyofindia.gov.in/Digital_Product_Show.aspx). The terms for usage can be viewed here: https://www.surveyofindia.gov.in/pages/copyright-policy.

India reported more than one million dengue cases and 2,712 deaths for the period, with the highest cases observed in 2021. 73% of the total cases occurred between 2016 and 2022, suggesting increased dengue outbreaks across the country. Overall, the fatality rate was 2.1 (per 1000 cases), the highest seen in 2007 (12.5). The northern region consisting of the hilly states of Himachal Pradesh, Jammu and Kashmir, and Uttarakhand, and the Gangetic plains of Punjab, Uttar Pradesh, Delhi and Chandigarh contributed the highest proportion (28.8%) of dengue cases among all the regions. The southern states of Andhra Pradesh, Goa, Karnataka, Kerala, Tamil Nadu and Telangana and the union territory of Puducherry were responsible for 26.2% of the cases. The northeastern states, historically part of the malaria belt [Dev & Manguin, 2021], reported ~60000 cases or 2.2% of the total dengue cases across India. The Western region defined by the three states – Maharashtra, Gujarat and Rajasthan and the union territories of Daman & Diu and Dadra and Nagar Haveli, accounted for 20.5% of the total cases but the highest share of deaths (31%) during the same period. The northern and southern states contributed 28.1% and 24.1% of deaths, respectively, while the eastern states attributed for 18.9% of the total cases. The zonal distribution of dengue cases and deaths is provided in Fig 3.

West Bengal reported the maximum number of dengue cases, followed by Maharashtra and Karnataka. The Northeastern states of Mizoram, Arunachal Pradesh, Sikkim, Nagaland, and Meghalaya contributed the least, varying between 0.04% and 0.2% of the total cases. The dengue cases were initially (i.e., 2007–2011) restricted to a few states only, such as the western states of Gujarat, Maharashtra, and Rajasthan, as well as all the southern states except Goa and Andhra Pradesh. In the north, the capital union territory of Delhi, Punjab and Uttar Pradesh, later after 2015, also reported higher cases as well as deaths, thus contributing to the higher case fatality rate observed.

### 3.2. Annual changes in dengue across the states

**Fig 4:**
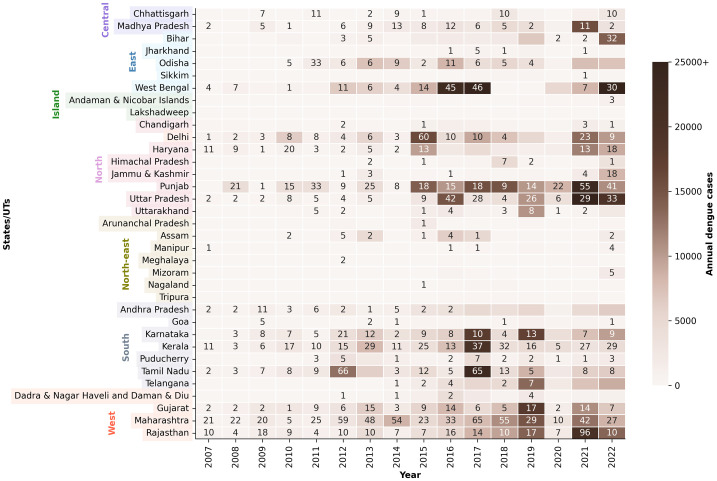
Spatiotemporal distribution of dengue cases and deaths across the Indian states and union territories between 2007 and 2022. The states have been grouped by the zones which is also shown in Fig 3. The numbers indicate the deaths, and the colours denote the cases.

The spatiotemporal changes in dengue cases and deaths are depicted in Fig 4. Between 2007 and 2011 (period 1), maximum dengue cases were reported from the northern states of Punjab and Delhi, accounting for 28% of the total cases reported. Low incidences were observed in states outside the main peninsular region, such as Jammu & Kashmir (5), Himachal Pradesh (3), Sikkim (2), Meghalaya (1) and Mizoram (1), and no cases were reported from the northeastern states of Arunachal Pradesh and Tripura. Compared to period 1(2847), dengue incidences in West Bengal increased by ~17-fold in period 2 (2012–2016; 47691) and ~ 28-fold in period 3 (2017–2022; 80701).

States like Mizoram (growth factor of 655), Jammu & Kashmir (417), Meghalaya (255), Himachal Pradesh (168) and Sikkim (74) reported increased outbreaks in period 2. It is interesting to note that these states belong to the Indian Himalayan region [Negi et al., 2021], which has relatively colder temperatures than the rest of the country. Despite these low temperatures, dengue cases have exponentially increased, which could be due to warming temperatures, as multiple studies have reported an increase of 0.1°C – 0.7°C in the mean surface air temperature for the Himalayan region in the past decade [55,56,57,58].

Further, these states attract millions of both domestic and international tourists, which has increased by 270 times between 2001 and 2021, while accounting for 22% of the state’s Gross Domestic Product [59]. Previously, in Bali (Indonesia), local tourism was positively associated with dengue cases, while urgent measures for climate and tourism integration were advocated [60]. An association was also observed between the annual disease incidence rate and the infection rate in travellers returning to Europe from dengue-affected countries [61]. Thus, the role of tourism in conjunction with the warming temperatures cannot be ruled out for the increased outbreaks in these high-altitude regions of the Indian subcontinent. Recently, a systematic literature synthesis reported the geographic expansion of vector-borne diseases into the Hindu-Kush region, which was considered non-endemic in the past [62].

Dengue in Himachal Pradesh followed a rising trend with a growth of 1625% in period 3 when compared to period 2. Manipur and Sikkim also saw higher cases in period 3, with an increase of 845% and 766%, respectively. The proportionate rise in the northern regions of Haryana, Delhi and Chandigarh was lower in period 3 than in period 2. The decline in dengue was reported in Tamil Nadu, Odisha, Kerala, Puducherry, Assam and Arunachal Pradesh. For instance, dengue cases in Assam and Arunachal Pradesh decreased by ~80% and ~90%, respectively, in period 3 compared to period 2.

The overall case fatality, i.e., the ratio of reported deaths and cases, was 2.1 per 1000 cases for the entire country. Only 524 deaths were reported between 2007 and 2012, while the deaths in period 2 (1037) and period 3 (1043) were comparable; the case fatality was relatively higher in period 2 (2.6 per 1000) than in period 3 (1.4 per 1000). Maharashtra accounted for the maximum number of deaths, with a total of 473 deaths reported between 2007 and 2022. The higher numbers might be attributed to the well-established cause-specific death reporting system in Maharashtra, which is one of the most efficient across India [63] About ~80% of the burden due to deaths was reported from Maharashtra, Punjab, Kerala, Rajasthan, Uttar Pradesh, Tamil Nadu, Karnataka, Gujarat, West Bengal and the national capital territory of Delhi.

The three-year moving averages and dengue cases are provided in Fig A in S1 Appendix. For the islands of Andaman and Nicobar in the Bay of Bengal and Lakshadweep in the Andaman Sea, an upward trend was observed after 2018. These regions have witnessed many tourists from mainland India in recent years [64]. Post-2014, Andhra Pradesh, Goa and Telangana have reported an upward trend in cases; the latter saw a downward trend after the COVID-19 pandemic. Between 2010 and 2019, most of the northeastern states of Arunachal Pradesh, Assam, Mizoram, Nagaland, and Tripura reported an upward trend for most of the years. The eastern states also depicted similar characteristics; however, a downward trend was observed after the pandemic. States such as Odisha, Delhi, Chandigarh, Haryana, Jammu & Kashmir, Uttar Pradesh, Rajasthan and Maharashtra have observed a consistent upward trend.

### 3.3. Impact of climate, land-use, land-cover and socio-economic inequities

#### 3.3.1. Description of the identified determinants and their implications.

**Fig 5:**
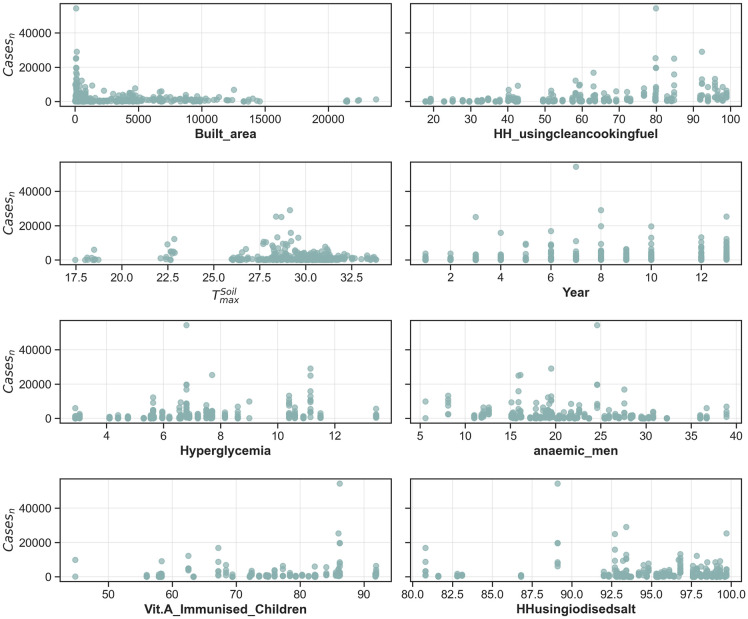
Comparison of normalised reported cases (Cases_n_) and distribution of covariates identified using the supervised regression model.

The supervised stepwise regression model had an adjusted R^2^ of 0.61, while the selected socio-economic variables for the prediction model and their coefficients are provided in Table 2, and the distribution of normalised cases with the selected covariates is given in Fig 5. The Variance Inflation Factor or VIF (Fig C in S1 Appendix), the effect of quantile transformation (Fig D in S1 Appendix), and the distribution of residuals (Fig B in S1 Appendix) are provided as supplementary figures. The VIF for the identified potential covariates were less than 1.5, which suggests that multicollinearity is absent. Moreover, the regression results are reliable, and the determinants identified here are independently contributing, which allows for clear effect interpretation [65]. We also obtained significant Kolmogorov-Smirnov and Jarque-Bera statistics that indicate no heteroskedasticity and normality of residuals. This is also suggested by the spread of residuals along the predicted logarithmic scale of cases. The absence of any visible patterns and normality of residuals indicates that the regression model satisfies the key validity assumptions, which ensures unbiased and trustworthy estimates of p-values and confidence intervals [66].

**Table 2:** Significant variables (p-value < 0.05) identified from the regression model and their magnitude of dependence. (Met.: Meteorological, SE: Socio-economic, and LU/LC: Land-use Land-Cover).

Variables	Type	Coefficient
*Built area*	LU/LC	0.86
*Max Soil Temperature*	Met.	0.54
*Year*		0.19
*Prevalence of Hyperglycaemia*	SE	0.26
*Prevalence of Anaemia in men*	SE	0.31
*Proportion of children immunised with Vitamin A*	SE	-0.24
*Proportion of households using iodised salt*	SE	-0.16
*Proportion of households using clean cooking fuel*	SE	0.49

We note that the built areas which is representative of urbanisation, as having the highest magnitude of dependence. Dengue cases in the past have been previously associated with urbanisation and population density [67,68]. Previously, higher seroprevalence was observed in the urban areas (70.9%) compared to the rural areas (42.3%), thus further indicating that urbanisation is among the primary drivers for increased dengue incidences as discussed above [30,10]. However, the spread to rural areas should not be overlooked, as multiple recent outbreaks have been reported that were attributed to enhanced transport mobility, expansion of peri-urbanisation, and improved diagnosis [9]. Additionally, rural areas in India present significant challenges in healthcare distribution, resulting in inequitable access to and utilization of healthcare services [69,70].

Among the meteorological variables, Soil T_max_ reported the highest magnitude of dependence. In India, the maximum soil temperature was observed during summers, while previously multiple studies have reported a convergence between the delayed impact of weather parameters such as Soil T_max_ observed in the pre-monsoon period and the later dengue outbreaks [71,72,73]. However, we are limited by the annual data in measuring the true impacts of seasonality on the cases, while the increase in cases during post-monsoon months has been reported in multiple locations across India [74,75,76].

The supervised model also indicates a positive dependence on the Year variable, suggesting that the dengue cases will increase in the future. The proportion of children immunised with Vitamin A, the proportion of households using iodised salt, and clean cooking fuel were the socio-economic covariates, and they were identified as potential determinants of dengue transmission across states using the supervised regression model. The dietary intake of iodised salt has a significant role in normal physical growth and development at all stages of life, from childhood to adulthood [77] Back in 1962, India launched a salt iodisation programme named the National Iodine Deficiency Disorders Control Programme for the prevention and control of Iodine Deficiency Disorders [78]. The program’s success is evident in the National Iodine and Salt Intake Survey conducted in 2014–15, which reports a 91.7% household coverage of iodised salt [79]. The recent NFHS-5 reported 93% of households to be using iodised salt; however, Andhra Pradesh (83%) and the union territory of Dadra & Nagar Haveli and Daman & Diu (89.1%) were among the states having relatively lesser coverage. Thus, a fortified diet including iodised salt and other essential nutrients can lead to better immunity, which could reduce the severity of dengue and susceptibility towards other infectious diseases.

In India, there is wider accessibility of clean fuel, especially in urban areas (89.7%) compared to rural areas (43.2%), according to the NFHS-5. In 2016, the Union Government of India introduced a targeted campaign named the Pradhan Mantri Ujjwala Yojana that provided subsidised liquefied petroleum gas to poorer sections of society to adopt cleaner fuel for cooking [80]. The observed positive dependence could be indicative of the higher prevalence in urban areas, which also have higher accessibility to clean fuel. Moreover, the households using clean cooking fuel are likely indicative of the obesity prevalence in both men and women, as a strong, significant Spearman’s correlation (r-value > 0.8) was observed between the obesity prevalence in both genders and households using clean fuels for cooking. Previously, multiple studies have reported a convergence between increased risk of dengue and obesity [81,82].

In addition, Vitamin A is an essential micronutrient that is linked to vision, immunity and survival [83]. Its deficiency has been associated with impaired humoral and cell-mediated immunity that could lead to other complications [84]. The public health problem associated with the deficiency of Vitamin A is well documented in India despite the National Prophylaxis Programme against Nutritional Blindness due to Vitamin A that was initiated in 1970 [83,85]. From the recently conducted NFHS-5, only 36.8% of children aged 6–59 months across India were given Vit. A supplement, while 47% had food rich in Vitamin A at the time of the survey. This proportion was lower in the eastern and northeastern states such as Bihar (30.4%), Manipur (20.3%) and Nagaland (21.4%). Previously, in Guatemala, an association between dengue and lower levels of both β-carotene and retinol was reported [86]; however, little research exists on their associations [84].

Among the health indicators, a positive dependence was reported between the state-wise reported dengue transmission and the prevalence of hyperglycemia and anaemia. Both hyperglycemia and anaemia are reported to be highly comorbid with severe dengue [87,88]. In India, 21.6% of men aged between 15–49 years are reportedly diagnosed with anaemia according to the NFHS-5, with states like West Bengal, Tripura, Jammu & Kashmir, Assam, Jharkhand, Odisha, Chhattisgarh and Gujarat reporting prevalence > 25% of the total. Further, glucose was linked to a significant increase in DENV genomic levels in *Aedes* mosquitoes feeding on blood meals, while it also facilitates faster viral transmission [89]. Around 7.2% of the population was affected with hyperglycemia as per the NFHS-5, whereas 15.6% have been prescribed anti-diabetic drugs for controlling their blood glucose levels. This proportion was, however, higher in the urban areas than in the rural areas.

#### 3.3.2. Inferences from the ensemble model.

**Fig 6:**
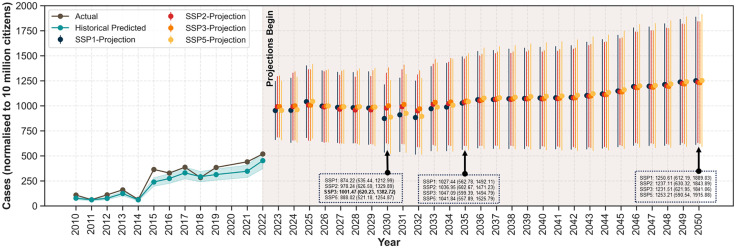
Comparison plot between year-wise actual and predicted cases for the whole country, normalised to 10 million citizens. The error bar represents the uncertainties of the predictions across the states.

We report that the selected ensemble model achieves a prediction accuracy of 91.7%, with a final mean absolute error of 17.76%. This error accounts for uncertainties in the input data and the mean prediction error from 100 independent Monte Carlo simulations of the ensemble model. The historical analysis suggests that the model underestimated the normalised cases across the years, except for 2011, 2014 and 2018. The comparison between the year-wise actual and predicted cases for the whole country is provided in Fig 6.

The predictions indicate that India is expected to witness a consistent increase in dengue infections in all scenarios, irrespective of socio-economic targets. We observe that in 2030, a development focused on SSP3 (1001.47; 95% CI: 620.23, 1382.72) is projected to have the highest normalised cases per 10 million, followed by SSP2 (978.24; 95% CI: 626.59, 1329.89). Both SSP1 (874.2; 95% CI: 535.4, 1212.9) and SSP5 (888.02; 95% CI: 521.2, 1254.9) have a relatively lesser burden.

In 2035, a SSP3 mode of development is projected to have the maximum burden (1047.1; 95% CI: 599.4, 1494.8), followed by a fossil-fueled development (1041.84; 95% CI: 557.9, 1525.8). SSP1 or the sustainability mode of development will have the minimum burden with a projected 1027.4 cases per 10 million (95% CI: 562.8, 1492.1). However, in the mid scenario, i.e., in 2050, both SSP1 (1250.61; 95% CI: 612.19, 1889.03) and SSP5 (1253.21; 95% CI: 590.54, 1915.88) could lead to the highest national dengue burden.

The averaged feature importance, which explains the overall contribution of each determinant to the predictions of the ensemble model, suggests that per capita income is the highest contributing variable (29.2%), followed by year (16.3%), prevalence of hyperglycaemia (12.5%) and percent of households using clean cooking fuel (12.2%). We observe that the climatic variables accounted for between 5%-6.5% of the overall averaged feature importance. This suggests that socio-economic and health covariates should be included in prediction models, along with climatic variables.

**Fig 7:**
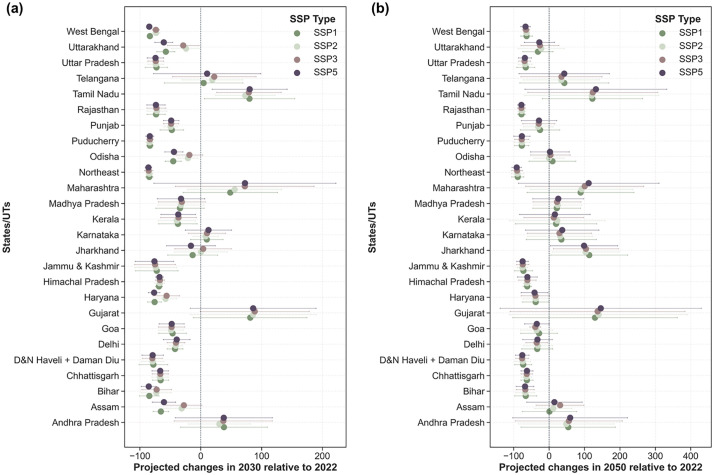
Projected changes in the dengue cases w.r.t reported cases in 2022, corresponding to different socio-economic shared pathways in (a) 2030 and (b) 2050. The dots represent the mean % projected changes; the lines represent the confidence intervals. SSP1: Sustainability, SSP2: Middle of the Road, SSP3: Regional Rivalry, SSP5: Fossil-fueled Development.

The projected changes in annual normalised cases for each state relative to 2022 and corresponding to different SSPs in 2030 are shown in Fig 7a. For the near scenario, i.e., in 2030, the relative changes could be differential across the states. We observe that the southern states of Karnataka, Andhra Pradesh and Tamil Nadu are projected to have an increase between 10–80%, whereas the states of Kerala and Goa will have a relative decrease. In contrast, the western states of Gujarat and Maharashtra are expected to have the maximum proportional increase in 2030. This trend was also observed between 2016 and 2022 (Phase 2). These states are projected to experience significant urbanisation, population growth, and rising per-capita income [90,51].

Under the SSP1 mode of development, a maximum relative decrease and the minimum relative increase are projected for ~75% of the states. As discussed earlier, SSP5 is also leading to a relatively lesser increase and a higher decrease. Although development that follows an SSP3 scenario is going to witness the maximum burden, SSP1 or sustainability is the scenario that will see fewer outbreaks.

In the northern Gangetic plains spanning across the states of Uttar Pradesh, Bihar, Haryana, and West Bengal, a relative decrease in estimated cases is projected. Presently, the Gangetic basin is home to ~50% of the country’s population and consequently has a very high population density and significant economic activity [91]. For instance, West Bengal may see a ~ 85% decrease in SSP1 and SSP5 scenarios. The reduction will be relatively less under SSP2 and SSP3. A similar trend is observed for the Rajasthan state, which is famous for the Thar Desert. Similarly, a relative reduction is projected among the hilly forested states in the north and northeastern India.

The projected proportional change in the dengue cases for the mid scenario, i.e., 2050, is provided in Fig 7b. Between 2030 and 2050, we estimate an increase in dengue cases in most of the states. In the eastern India, Jharkhand is projected to have very high relative increase varying between 100–120% for all SSP scenarios. The cases are projected to increase further in Gujarat, Maharashtra, Kerala, Karnataka, Andhra Pradesh, Telangana and Tamil Nadu. In the central state of Madhya Pradesh, which is traditionally a malaria endemic state, an increase of >20% is projected for all developmental scenarios. By the 2050s, both SSP1 and SSP5 modes of development are projected to have the maximum relative increase across the country which could be attributed to the improved health infrastructure and capacity building, thus ensuring better reporting.

Overall, we can estimate the occurrence of increased outbreaks and their magnitude across the country for different SSP scenarios. Clearly, SSP1 is the better alternative for the country. However, region-specific interventions need to be carried out, as the magnitude of the interventions is not uniform as expected for the diverse socio-economic, cultural and geographical aspects of the country. The sensitivity analysis for our ensemble model suggests that the model is particularly sensitive to the malaria endemic regions in Central India and northeastern India. Nevertheless, these regions have witnessed rising dengue outbreaks in the past decade (Fig 4) [Dev et al., 2015], along with frequent malaria outbreaks. While there could be differences in biting behaviour due to different mosquitoes involved in the transmission of malaria and dengue, the diagnosis for the latter was often inadequate in these places, which has considerably improved over the past few years [40]. However, the health and economic burden of dengue can be minimised with better testing, reporting and health infrastructure.

## 4. Discussion

Through this study, we analysed the spatiotemporal distributions of dengue cases across India. To the best of our knowledge, this study is the first attempt to design India-specific scenarios of socio-economic development that are impacted by climate change and assess their impact on dengue cases across the states using ensemble models. By using climatic projections for each river basin, we predict the dengue cases for different climatic zones, which vary across the river basins in India. This supports the previous recommendation by Mutheneni et al., which assessed the extrinsic incubation period or the virus life cycle inside the *Aedes* mosquito, across India and found that the temperature and precipitation are influential in virus development in different climatic zones [92].

We believe that our work will help the national stakeholders in decision-making and preparing for future scenarios. India has witnessed strong economic and population growth in the last decade. However, with increased urbanisation and population density, adequate strategies need to be formulated for upgrading the infrastructure and capacity building to inform the stakeholders at the grassroots level to prevent any major outbreak. Community engagement about the impacts of climate change, personal preventive measures and the significance of fortified diets are recommended in the emerging hotspots. The government should also immediately formulate targeted climate-resilient strategies based on modelled results and involve real-time monitoring of climatic and dengue surveillance programs.

We appreciate the current vector control measures implemented by the Government, however, there is an urgent need to strengthen and enforce them on weekly basis especially during rainy seasons. In the urban areas, open drainage should be replaced with covered sewers. Additionally, community actions such as covering water storage facilities, removing standing water, and implementing proper garbage disposal are recommended for reducing dengue outbreaks. The ensemble model also predicts an increase in dengue cases in Madhya Pradesh and Jharkhand that are historically classified as malaria belts; hence, there is a need for simultaneous testing for both malaria and dengue in both rural and urban India. Technology supported by social media can play a great role in raising awareness and disseminating information to the general public. The results presented here are generalisable for similar low-middle income countries (LMIC) which report similar vector species, socioeconomic hierarchy and are presently witnessing increased urbanisation. However, there is also an urgent need for international collaborations which involves cross-boundary engagement especially in South Asian countries, as associations between climate and the dengue virus strongly depend on local climate [93].

## 5. Conclusion

We report that India is witnessing increased outbreaks and geographical expansions, especially in the malaria-endemic regions. We identified that urbanisation continues to be one of the primary drivers of the increased transmission; however, the rural areas should not be ignored, as a few states with higher rural populations have reported high cases. We also predict that the dengue cases will continue to increase, as indicated by the ensemble models, but the impact of socio-economic development will be different across the states. Our predictions show that development based on Regional Rivalry, where there are high challenges to both mitigation and adaptation, could contribute to the highest proportional increase in the near scenario. In contrast, the development focusing on Sustainability could witness a relatively lesser increase because of a concerted focus on health infrastructure. However, the focus should also be on increasing healthcare accessibility among the vulnerable sections of society, especially in remote or high-terrain areas that are hard to access.

However, our analysis is limited by the annual data, due to which the true effects of the seasonality cannot be accounted for. Using more granular temporal data in future studies will result in stronger evidence for climatic impacts on the dengue transmission by precisely capturing the temporal variations, as seasonal biases may arise due to the smoothing from annually aggregated data, potentially hiding the peak transmission periods. Future work could incorporate time-series or cyclic spline methods for modelling seasonality, which will help in a better understanding of temporal patterns and their drivers. Our analysis was also constrained by the lack of entomological- and sero-surveillance and testing data needed to normalise the cases, as this data reflects the regional differences in health infrastructure. Additionally, future analyses could benefit from incorporating information on the distribution of testing and diagnostic networks throughout the country. The modest feature-to-sample ratio in the present study limits the overall model capacity and increases the risk of overfitting. The use of tree-based ensemble methods with embedded regularization, combined with careful hyperparameter optimization and cross-validation, reduces but does not fully eliminate this concern [94,95,96,97]. Future work using more granular spatiotemporal data at weekly temporal and sub-district spatial scale will eliminate this concern, resulting in more accurate predictions. This way, the delayed impacts of weather conditions, socioeconomic and health conditions can also be assessed. We believe that the insights from our study will aid in setting up for subsequent cross-sectional and longitudinal studies to systematically evaluate the critical covariates contributing to increased vulnerability to dengue across the country.

## Supporting information

S1 Appendix Table A Indicators considered for the study. Table B Description of the SSPs considered for the study. These SSPS have been summarised in Riahi et al. [47]. Fig A 3-year moving average trends for dengue cases. Fig B Distribution of the residuals obtained from supervised association model. Fig C VIFs estimated for each of the covariates in the selected association model. Fig D Quantile-transformed data for the selected covariates.
(DOCX)
