# Supplementary material for: Impact of projected climate and socioeconomic scenarios on state-wise annual dengue incidence in India using ensemble models
Source: PLoS Negl Trop Dis. 2026 Mar 31;20(3):e0014159. doi: 10.1371/journal.pntd.0014159 (PMC13099098; doi:10.1371/journal.pntd.0014159)
Supplement: S1 Appendix — Table A Indicators considered for the study. Table B Description of the SSPs considered for the study. These SSPS have been summarised in Riahi et al. [47]. Fig A 3-year moving average trends for dengue cases. Fig B Distribution of the residuals obtained from supervised association model. Fig C VIFs estimated for each of the covariates in the selected association model. Fig D Quantile-transformed data for the selected covariates. (DOCX) [file pntd.0014159.s001.docx]

**S1 Appendix** on

***Impact of Projected Climate and Socioeconomic Scenarios on State-wise Annual Dengue Incidence in India using Ensemble Models***

**Supplementary Tables:**

Table A Indicators considered for the study.

| **Indicators** | **Parameters (Examples)** |
| --- | --- |
| Nutritional | Anaemia |
|  | BMI |
|  | Body structure ratio |
|  | Eating habits |
| Health | Blood Sugar level |
|  | Diarrhoea |
|  | Access to private/public health care |
|  | Vaccination accessibility |
|  | Tobacco usage |
|  | Blood Pressure |
|  | Mortality and birth rates |
|  | Underlying respiratory infections |
| Household | Men/ Women/ Children Education |
|  | Household facilities such as electricity etc. |
|  | Household cooking fuel |
|  | Household economic status |
|  | Women Empowerment |

Table B Description of the SSPs considered for the study.

| **Socioeconomic Shared Pathways** | **Mitigation Challenges** | **Adaptation Challenges** |
| --- | --- | --- |
| SSP1 (Sustainability) | Low | Low |
| SSP2 (Middle of the Road) | Middle | Middle |
| SSP3 (Regional Rivalry) | High | High |
| SSP5 (Fossil-fueled Development) | High | Low |

More information about these SSPs can be found in Riahi et al. (2017)

**Supplementary Figures:**


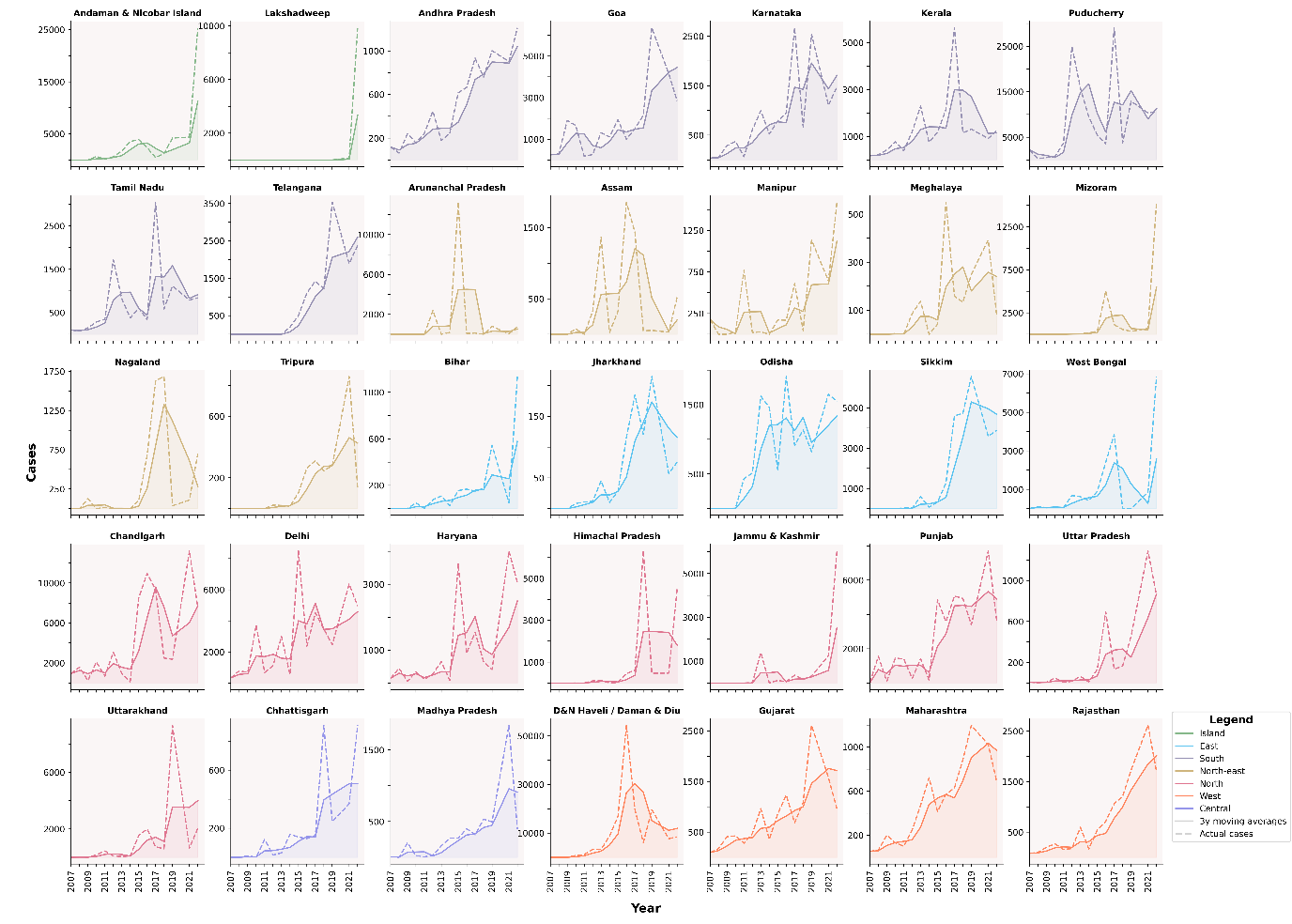


Fig A 3-year moving average trends for dengue cases


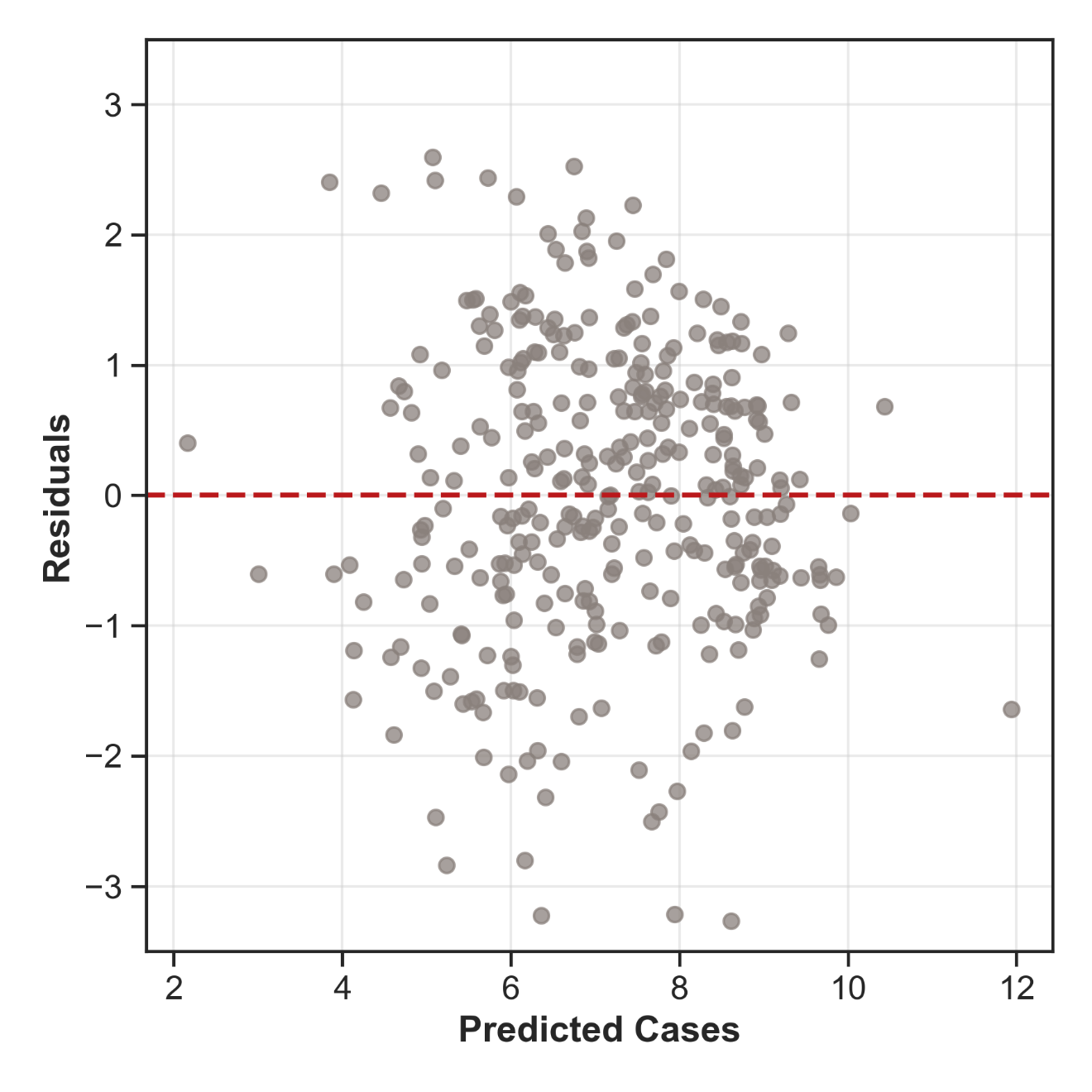


Fig B Distribution of the residuals obtained from supervised association model.


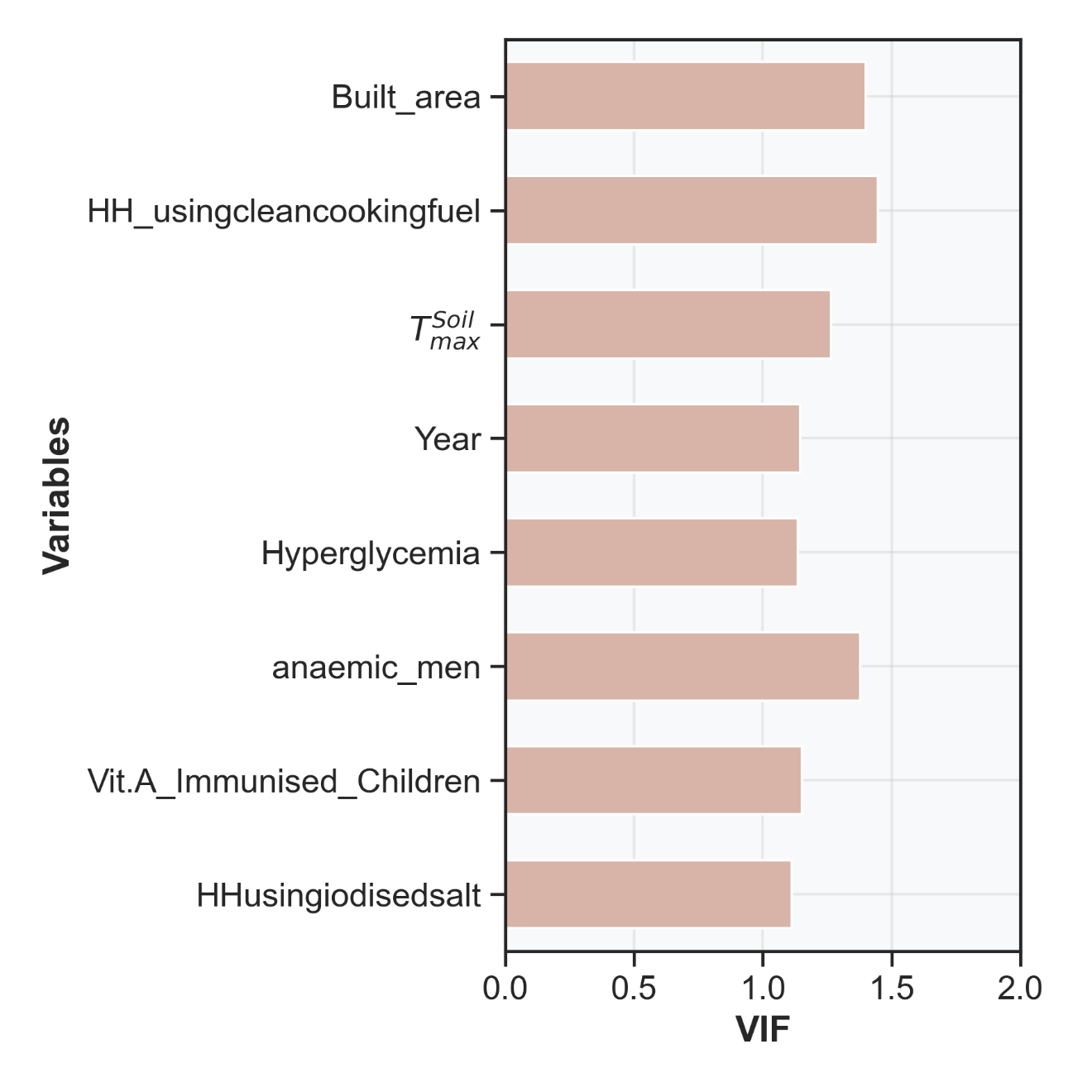


Fig C VIFs estimated for each of the covariates in the selected association model


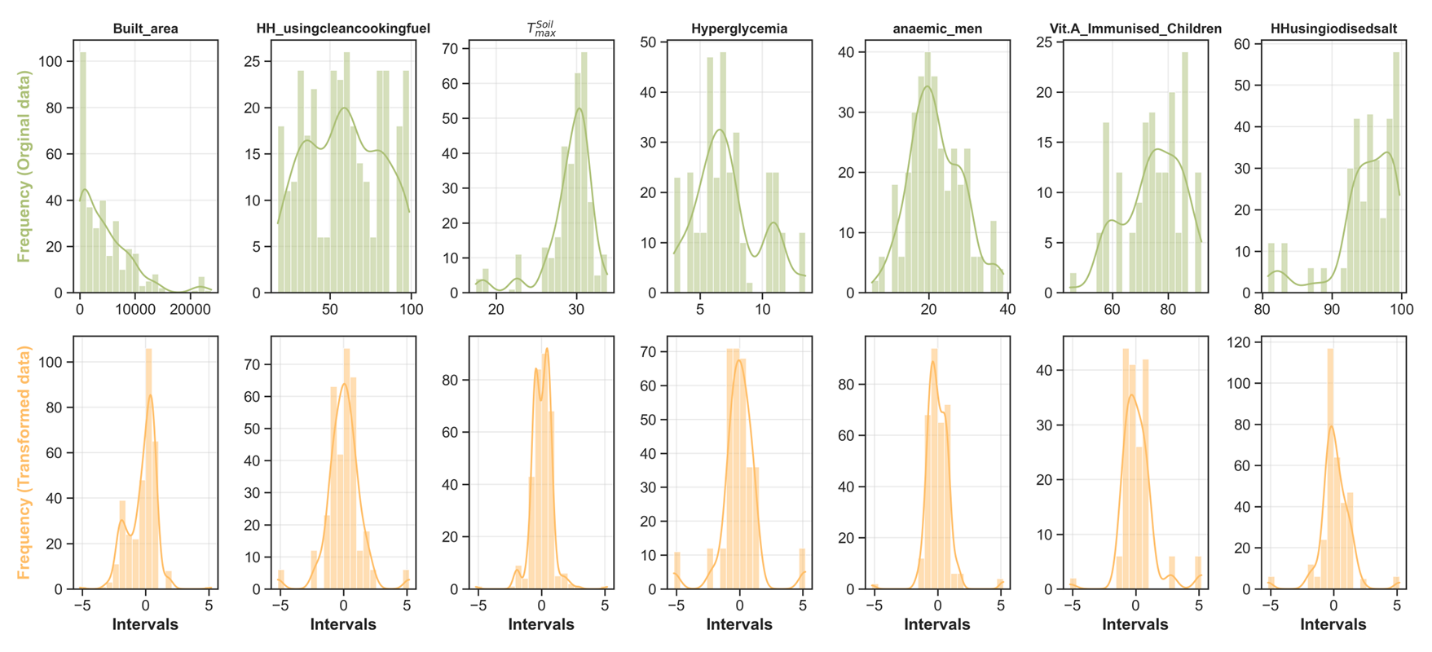


Fig D Quantile-transformed data for the selected covariates.
